# Delayed Radiological Resolution: A Comparative Longitudinal Study of Viral Pneumonia Evolution in Diabetic vs. Non-Diabetic Patients

**DOI:** 10.3390/diseases14060210

**Published:** 2026-06-10

**Authors:** Ana Maria Mihai, Ovidiu Rosca, Florina Lucaciu, Alexandra Herlo, Talida Georgiana Cut, Ioana-Melinda Luput-Andrica, Radu Gheorghe Dan, Matilda Radulescu, Andreea Cristina Floruncut, Adelina Marinescu, Alexandra Sima

**Affiliations:** 1 Doctoral School, “Victor Babes” University of Medicine and Pharmacy Timisoara, Eftimie Murgu Square 2, 300041 Timisoara, Romania; ana-maria.mihai@umft.ro (A.M.M.);; 2 Department XIII, Discipline of Infectious Diseases, “Victor Babes” University of Medicine and Pharmacy Timisoara, 2 Eftimie Murgu Square, 300041 Timisoara, Romania; alexandra.mocanu@umft.ro (A.H.);; 3 Victor Babes Clinical Hospital for Infectious Diseases and Pneumology of Timisoara, 300041 Timisoara, Romania; 4 Center for Hepato-Biliary-Pancreatic Surgery, “Victor Babeș” University of Medicine and Pharmacy, 300041 Timisoara, Romania; radu.dan@umft.ro; 5 Department of Surgery I, “Victor Babeș” University of Medicine and Pharmacy, 300041 Timisoara, Romania; 6 Department of Microbiology, Division of Microbiology, “Victor Babeș” University of Medicine and Pharmacy Timișoara, Eftimie Murgu Square No. 2, 300041 Timisoara, Romania; 7 Second Department of Internal Medicine, “Victor Babes” University of Medicine and Pharmacy, 300041 Timisoara, Romania; sima.alexandra@umft.ro; 8 Department of Diabetes, Nutrition and Metabolic Diseases Clinic, “Pius Brînzeu” Emergency Clinical County University Hospital, 300723 Timisoara, Romania

**Keywords:** viral pneumonia, diabetes mellitus, chest radiography, radiological lag, lethal split, attrition bias, risk stratification, pulmonary clearance, hyperglycemia, prognosis

## Abstract

Background: Diabetic patients face increased severity in viral respiratory infections, yet during the longitudinal progression of lung recovery, the radiological clearance is poorly quantified. Methods: This prospective study at an infectious diseases hospital analyzed 430 patients with confirmed viral pneumonia. Radiological severity (Rx) and pleural effusion were scored (0–3) at admission (day 1) and follow-up (day 6). Results: Diabetics presented with significantly higher baseline severity (Median 2.0 vs. 1.0, *p* < 0.0001). While both groups improved at identical rates (Median Δ = −1.0), a significant radiological lag persisted in diabetics at day 6 (Median 1.0 vs. 0.0, *p* < 0.0001). Attrition analysis (N = 75) revealed a divergent lethal split: attrition in the non-diabetic cohort was almost exclusively driven by low-severity early departures against medical advice (94.2%), whereas diabetic attrition was primarily characterized by early mortality (60.9%; *p* < 0.0001). Although the diabetic state was associated with a pronounced radiographic resolution delay in unadjusted comparisons, this disadvantage was substantially attenuated and lost statistical significance after adjustment for admission radiological severity (adjusted OR 2.04, 95% CI 0.88–4.76) and chronic comorbidity burden (adjusted OR 1.37, 95% CI 0.56–3.39), indicating that the diabetic lag is largely explained by a higher presenting severity and comorbidity burden rather than by an independent acute effect of diabetes itself. Sensitivity analyses suggest that the observed lag in survivors likely underestimates the true disease burden, given the concentration of early mortality among high-risk diabetic cases.

## 1. Introduction

Lung diseases remain a global health burden, contributing significantly to worldwide morbidity and mortality [1,2,3]. Among the most challenging aspects of clinical management is the heterogeneity of recovery in viral pneumonia [4]. While biochemical markers often guide the treatment, they frequently fail to reflect the physical resolution of pulmonary damage, a phenomenon we term radiological lag. Diabetes mellitus has long been suspected of slowing clinical recovery [5,6,7,8,9,10,11,12], but its impact on the longitudinal evolution of lung scars remains under-researched [5,13,14,15,16]. Recent diagnostic advances emphasize the need for better phenotyping and risk stratification [7]. This prospective study aims to investigate the metabolic link between hyperglycemia and delayed lung clearance, while specifically addressing the impact of patient attrition on study outcomes through the analysis of the lethal split.

Translating epidemiological insights into effective management strategies remains a priority in pulmonary medicine, particularly for heterogeneous conditions where resolution patterns vary by metabolic phenotype. Beyond individual clinical outcomes, the inability of diabetic patients to reach radiological resolution within standard timeframes imposes a significant burden on healthcare utilization, necessitating longer hospital stays and more intensive respiratory support [16,17,18].

The clinical consequences of a delayed radiological resolution extend well beyond the inpatient setting. Persistent radiographic abnormalities at the conclusion of standard antiviral therapy may obscure the true point of physiological recovery, complicate discharge decisions and contribute to premature de-escalation of monitoring in patients who remain biologically vulnerable. A residual pulmonary lesion represents incompletely cleared parenchymal injury, a state that has been linked in contemporary cohorts to a higher risk of secondary bacterial infection, readmission and protracted functional impairment. In diabetic population specifically, the convergence of impaired innate immunity, microvascular compromise and dysregulated tissue repair plausibly prolongs the window of radiological vulnerability, yet the magnitude and time-course of the effect have rarely been quantified using paired imaging in a real-world hospitalized cohort. Characterizing this lag is therefore not merely a descriptive exercise; it carries direct implications for the timing of follow-up imaging, the duration of inpatient observation and the allocation of advanced respiratory support.

Against this background, the present study was designed to address three specific gaps. First, it quantifies the longitudinal trajectory of radiological severity in diabetic versus non-diabetic patients using standardized paired chest radiography at admission and at the end of the primary therapeutic phase. Second, it explicitly characterizes the pattern of patient attrition, distinguishing early mortality from early discharge, in order to assess the direction and likely magnitude of survivor bias rather than treating dropout as simple missing data. Third, it evaluates whether the diabetic state, as opposed to individual acute or chronic glycemic indices, independently predicts the failure to achieve radiological improvement. By integrating these aims, this work seeks to provide a clinically actionable description of radiological lag and to clarify the extent to which it may be underestimated when the most severe cases are lost before follow-up imaging.

## 2. Materials and Methods

### 2.1. Study Design and Setting

This prospective, longitudinal study was conducted at a tertiary infectious diseases hospital from December 2024 to April 2026. The study aimed to evaluate the impact of diabetes mellitus on the radiological resolution of viral pneumonia in hospitalized patients. The ordinal scoring system was developed internally to provide a parsimonious, clinically applicable metric for longitudinal assessment.

### 2.2. Patient Selection and Ethical Considerations

The study protocol was approved by the institutional Ethics Committee and internal review board (Approval No. 12066/12 December 2024). All participants provided written informed consent prior to enrollment.

- Inclusion Criteria: Adults (≥18 years) hospitalized with clinical signs of respiratory tract infection and a confirmed positive PCR diagnosis for one or more of the following viral pathogens: Influenza A (A/H1, A/H3, AH1-2009), Influenza B, Adenovirus, Respiratory Syncytial Virus (RSV), Human Metapneumovirus (hMPV), Human Rhinovirus, Parainfluenza 1–4, MERS-CoV, SARS-CoV-2 and Coronavirus (229E, HKU1, NL63, OC43)
- Exclusion Criteria: Patients under the age of 18 or those who failed to provide informed consent.

### 2.3. Radiological Scoring and Follow-Up

To align with real-world clinical workflows and optimize patient care, baseline imaging at admission (day 1) utilized standard digital chest X-rays. The X-ray results were processed through a parsimonious, internally developed macro-structural ordinal scale (0–3) focusing on gross anatomical distribution: 0 = Normal; 1 = Minimal interstitial findings; 2 = Unilateral pneumonia; and 3 = Bilateral bronchopneumonia/ARDS. At follow-up (day 6), all surviving patients were evaluated uniformly using standard digital chest X-rays to assess early radiological evolution while minimizing cumulative radiation exposure.

Findings were scored using a standardized ordinal scale (0–3):

- Parenchymal Involvement (Rx): 0 = Normal; 1 = Minimal interstitial findings; 2 = Unilateral pneumonia; 3 = Bronchopneumonia and ARDS.
- Pleural Effusion: 0 = No liquid; 1 = Minimal; 2 = Moderate; 3 = Large amount.
- Recovery Metrics: The speed of recovery was calculated as Rx_Delta = Rx_6 − Rx_1.

The follow-up scan at day 6 was specifically timed to coincide with the completion of standard 5-day antiviral protocols (e.g., for SARS-CoV-2 and Influenza). This interval allows for an objective assessment of pulmonary resolution at the conclusion of the primary therapeutic phase, providing a snapshot of clinical readiness for discharge or the need for continued inpatient care.

All radiographs were scored independently by two assessors, a senior infectious-diseases physician and a pulmonologist, each blinded to the patient’s diabetic status, laboratory results, and the timing (day 1 versus day 6) of the film. Disagreements between the two readers were resolved by consensus discussion. Formal inter-reader reliability statistics (e.g., weighted Cohen’s κ) were not computed in the present study and are planned as part of a forthcoming validation analysis of the ordinal scale. The parenchymal involvement score (Rx) and the pleural effusion score were assigned separately because they reflect distinct pathophysiological processes, alveolar/interstitial injury and fluid accumulation, and may evolve at different rates. For the purposes of the regression analysis, “failure to radiographically improve” was defined a priori as a day 6 parenchymal score that was equal to or greater than the admission score (Rx_Delta ≥ 0), denoting the absence of any measurable reduction in radiological severity over the observation window.

### 2.4. Therapeutic Protocol

All patients received standard-of-care treatment according to local guidelines for SARS-CoV-2 and Influenza virus. For other viral pathogens, standardized supportive treatment was administered. To minimize therapeutic confounding, corticosteroid administration was standardized. Both cohorts received identical low-to-moderate dose regimens according to current clinical guidelines; no high-dose pulse therapy was utilized. This standardization ensures that the observed radiological lag and metabolic markers were not skewed by disparate steroid exposure between the diabetic and non-diabetic groups.

### 2.5. Statistical Analysis

Statistical analyses were performed using MedCalc^®^ version 23.5.5 (MedCalc Software Ltd., Ostend, Belgium) [19]. Data normality was assessed using the D’Agostino–Pearson test. Continuous and ordinal variables were non-normally distributed and are presented as medians with interquartile ranges (IQR). Baseline and follow-up clinical and radiographic characteristics between independent cohorts (diabetic vs. non-diabetic) were compared using the Mann–Whitney U test. Longitudinal intra-group changes in radiographic severity scores from admission (day 1) to follow-up (day 6) were evaluated using the Wilcoxon signed-rank test for paired samples.

Categorical data and cross-tabulations were evaluated using the Chi-squared test, and the Chi-squared test for trend was applied where appropriate. Attrition bias (“the lethal split”) was formally evaluated by comparing the baseline characteristics and clinical outcomes of patients lost to follow-up against the remaining longitudinal analytical cohort.

To identify independent predictors for the failure to radiographically improve by day 6 among survivors (N = 355), a unified multivariable logistic regression model was constructed using the forced-entry method (Enter). The model calculated adjusted odds ratios (OR) along with their corresponding 95% confidence intervals (CI). The regression model adjusted simultaneously for demographic characteristics (Age, BMI), primary metabolic phenotype (Diabetes Status) and acute/chronic glycemic markers (SHR_z3 and HbA1c). The overall fit of the logistic regression model was evaluated using model chi-squared statistics, and its discriminative capacity was cross-verified via Receiver Operating Characteristic (ROC) curve analysis to determine the Area Under the Curve (AUC). All statistical tests were two-tailed, and a value of *p* < 0.05 was considered statistically significant.

Because death and discharge against medical advice represent competing outcomes rather than data that are missing at random, the restriction of the primary longitudinal analysis to survivors with complete imaging carries a risk of survivor bias. To gauge the robustness of the radiological-lag finding to this attrition, two sensitivity analyses were performed. In a worst-case analysis, every patient who died before day 6 was assigned the most severe day 6 parenchymal category (Rx = 3), and the between-group comparison was repeated; in a best-case analysis, the low-severity early departures (discharged against medical advice with a clear admission film) were assigned a day 6 score of 0. In addition, a composite adverse endpoint, comprising death, persistent radiological abnormality at day 6, or absence of improvement, was constructed to incorporate the early-mortality cases that the survivor-only analysis necessarily excludes.

To address potential confounding by presenting severity and overall comorbidity burden, two additional multivariable models were constructed. Table 3 added the admission radiological score (RX_1) to the predictors of Table 2. Table 4 added a chronic comorbidity count, defined as the sum of organ-system involvement across eight pre-specified domains (musculoskeletal, neurological, cardiovascular, respiratory, digestive, urinary, endocrine, and hematological comorbidities). The direction, magnitude, and significance of the diabetes term were reported across all three models (Tables 1–4), and model fit was compared using the Akaike Information Criterion (AIC).

## 3. Results

### 3.1. Patient Disposition and the Lethal Split

**Figure 1:**
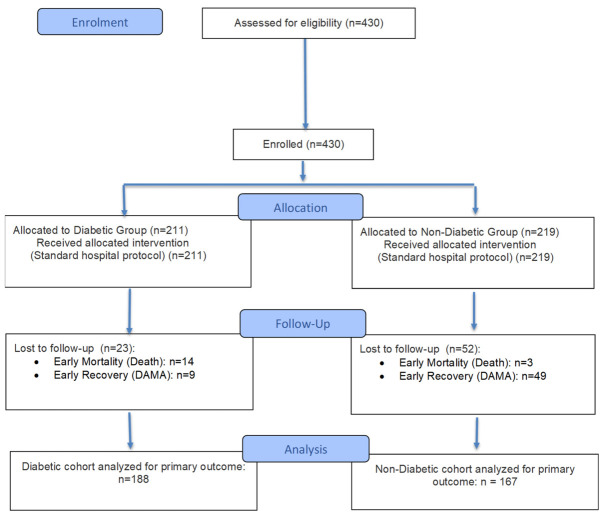
Flow Diagram. Illustrates patient enrollment, allocation to diabetic/non-diabetic cohorts and reasons for attrition (Early Mortality vs. DAMA).

A total of 430 patients were enrolled: 211 in the diabetic group and 219 in the non-diabetic group (Figure 1). During the study period, 75 patients (17.4%) were lost to follow-up before the day 6 scan.

Analysis of attrition revealed a highly significant divergence, which we have termed the lethal split (x^2^ = 27.252, *p* < 0.0001; Table 1):

- Non-diabetic attrition (n = 52): predominantly attributable to discharge against medical advice (DAMA). 94.2% (n = 49) left against medical advice, and these patients had a low baseline radiological severity (median Rx 0.0). Because objective recovery parameters (oxygen status, fever resolution, physician-documented clinical outcome) were not uniformly captured at the point of self-discharge, this group is more accurately described as low-severity early departures than as confirmed clinical recoveries.
- Diabetic attrition (n = 23): primarily driven by severity. 60.9% (n = 14) suffered early mortality, and these patients presented with significantly higher baseline pneumonia (median Rx 2.0, *p* < 0.0001).

**Table 1:** Characteristics of Attrition (“The Lethal Split”). Comparison of baseline severity and outcomes for patients lost to follow-up, highlighting the divergence between low severity early departures in non-diabetics and early mortality in diabetics.

Variable	Lost Diabetics (n = 23)	Lost Non-Diabetics (n = 52)	*p*-Value
Admission Rx Score	Median 2.0 (Pneumonia)	Median 0.0 (Clear Lungs)	<0.0001
Early Mortality (Death)	n = 14 (60.9%)	n = 3 (5.8%)	<0.0001
Early Departure (DAMA)	n = 9 (39.1%)	n = 49 (94.2%)	<0.0001

This distinct divergence in attrition patterns indicates that dropouts in the two cohorts were driven by fundamentally opposing clinical endpoints. Consequently, to avoid survivor bias, these early outcomes were modeled separately, and the subsequent longitudinal analysis was restricted to the remaining surviving patients who completed both imaging intervals.

### 3.2. Baseline Severity in the Longitudinal Cohort (n = 355)

The final analysis included 188 diabetics and 167 non-diabetics who completed follow-up imaging. At admission, diabetic patients exhibited a significantly higher burden of disease:

- Radiological Score: Diabetics presented with a median Rx of 2.0 compared to 1.0 for non-diabetics (*p* < 0.0001).
- Pleural Effusion: Baseline fluid involvement was significantly higher in the diabetic cohort (*p* < 0.0001).

### 3.3. Longitudinal Evolution and the Radiological Lag

**Figure 2:**
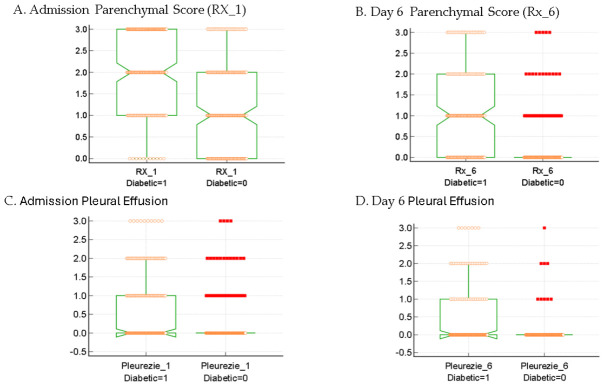
Longitudinal distribution of pulmonary involvement. (**A**) Baseline radiological severity (RX_1); (**B**) Follow-up severity at day 6 (Rx_6) illustrating the persistent radiological lag in survivors (*p* < 0.0001); (**C**) Baseline pleural effusion; (**D**) day 6 pleural effusion. Central notches represent the 95% Confidence Interval (CI) of the median; Note: Axis labels Pleurezie_1 and Pleurezie_6 denote pleural effusion states at admission and follow-up, respectively, preserved from primary clinical registry output definitions. Central notches represent the 95% Confidence Interval (CI) of the median; red squares indicate statistical outliers.

**Figure 3:**
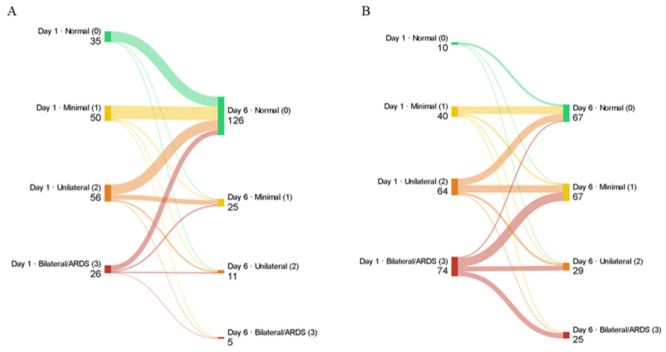
Alluvial diagram of patient flow between admission (day 1) and follow-up (day 6) parenchymal radiological score categories in the longitudinal survivor cohort (n = 355). (**A**) Non-diabetic patients (n = 167) demonstrate convergent flow toward radiological resolution, with 126 of 167 patients (75.4%) reaching a normal score (Rx = 0) by day 6. (**B**) Diabetic patients (n = 188) show persistent dispersion across all severity categories, with only 67 of 188 (35.6%) reaching Normal and 25 (13.3%) remaining at the Bilateral/ARDS level (Rx = 3), visually illustrating the radiological lag described in the text. Band thickness is proportional to the number of patients transitioning between categories. Node colors denote radiological severity: green = Normal, yellow = Minimal interstitial, orange = Unilateral pneumonia, red = Bilateral bronchopneumonia/ARDS.

A comparison of imaging findings between admission and the six-day follow-up demonstrated a significant disparity in recovery trajectories (Figure 2 and Figure 3).

- Radiographic improvement: Both cohorts showed statistically significant radiographic improvement over the study period (*p* < 0.0001 for both groups, Wilcoxon Signed-Rank test).
- The Healing Rate Paradox: The rate of change (Rx_Delta) did not differ significantly between groups. The median improvement for both diabetic and non-diabetic survivors was −1.0.
- The Persistent Gap: Despite similar improvement rates, the diabetic cohort failed to achieve clinical resolution. By day 6, the non-diabetic group had largely reached clear lungs (Median Rx: 0.0), while the diabetic group remained at a significantly higher severity level (Median Rx: 1.0) (*p* < 0.0001, Mann–Whitney U test; Figure 2).
- Pleural Effusion Stability: While the diabetic group showed higher initial pleural involvement (*p* < 0.0001), there was no significant difference in the evolution of pleural effusion (Pleural_Delta) between cohorts (*p* = 0.2442).

### 3.4. Clinical Correlation: Worsening Trajectories and Oxygen Support

While the majority of patients improved, a specific subset exhibited radiological progression:

- Deterioration Rate: Radiological worsening (Positive Rx_Delta) occurred in 11 diabetic patients (5.8%) compared to 7 non-diabetics (4.2%).
- Clinical Impact: In the diabetic cohort, all 11 patients who exhibited radiological worsening by day 6 required advanced oxygen therapy via High-Flow Nasal Oxygen or orotracheal intubation. This clinical correlation confirms that the observed radiological lag serves as a physical indicator of prolonged respiratory failure and increased healthcare utilization.

### 3.5. Predictors of Failure to Improve

**Figure 4:**
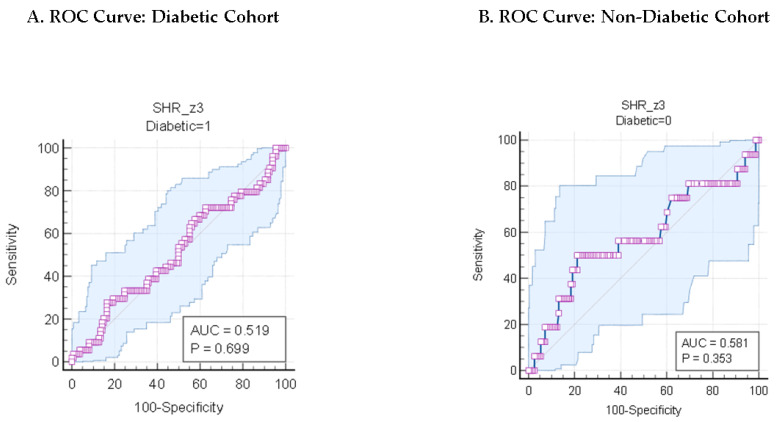
Receiver Operating Characteristic (ROC) curves for Stress Hyperglycemia Ratio (SHR_z3) as a predictor of radiological failure. (**A**) Diabetic cohort (AUC = 0.519); (**B**) Non-diabetic cohort (AUC = 0.581). In both groups, acute glycemic fluctuations showed poor discriminative power (*p* > 0.05), a pattern consistent with, though not proving, a binary-threshold interpretation in which the established diabetic state may exert a more uniform effect than do acute-phase glucose variations.

A unified multivariable logistic regression model was constructed to evaluate independent metabolic and demographic predictors of a failure to radiographically improve by day 6 within the surviving cohort (N = 355). In contrast to individual acute phase glycemic fluctuations (SHR_z3, *p* = 0.3281, Figure 4) or chronic glycated hemoglobin levels (HbA1c, *p* = 0.3990), which did not demonstrate independent predictive power, the clinical diagnosis of diabetes mellitus emerged as a highly significant independent predictor of non-improvement (Adjusted OR = 2.64, 95% CI: 1.17 to 5.97, *p* = 0.0196; Table 2). The overall model demonstrated excellent mathematical validity (Chi-squared = 25.355, df = 5, *p* < 0.0001). These findings are consistent with the diabetic state, rather than minor secondary glycemic fluctuations during acute hospitalization, being the dominant metabolic correlate of delayed pulmonary clearance in this cohort. When the model was additionally adjusted for the admission radiological score (Table 2 and Table 3), the diabetes term remained in the same direction but was attenuated to non-significance (adjusted OR = 2.04, 95% CI: 0.88–4.76, *p* = 0.098), while baseline RX_1 emerged as a strong independent predictor (OR = 2.31, 95% CI: 1.61–3.31, *p* < 0.0001). In a further sensitivity model additionally incorporating a chronic comorbidity count (Table 4), the diabetes effect attenuated further (OR = 1.37, 95% CI: 0.56–3.39, *p* = 0.49), with both baseline severity (OR = 2.12, *p* < 0.001) and comorbidity burden (OR = 1.45 per additional system involved, *p* < 0.001) emerging as independent predictors. These findings suggest that the diabetic disadvantage observed in unadjusted comparisons is substantially mediated by higher presenting severity and a greater chronic disease burden, rather than reflecting an independent intrinsic effect of the diabetic state on radiological clearance kinetics during the acute phase.

**Table 2:** Multivariable Logistic Regression Predictors for the Failure to Radiographically Improve (N = 355). Note: Overall Model Fit Chi-squared = 25.355, df = 5, Significance level *p* < 0.0001.

Independent Variable	Coefficient	Standard Error	Adjusted Odds Ratio (95% CI)	*p*-Value
Diabetic Status (Ref: Non-Diabetic)	0.9709	0.4160	2.64 (1.17 to 5.97)	0.0196
Age	0.0187	0.0118	1.02 (1.00 to 1.04)	0.1110
BMI	−0.0014	0.0183	1.00 (0.96 to 1.04)	0.9403
SHR_z3	0.2834	0.2898	1.33 (0.75 to 2.34)	0.3281
HbA1c	0.0777	0.0921	1.08 (0.90 to 1.29)	0.3990

**Table 3:** Adjusted for admission Rx_1; Overall model: χ^2^ = 50.062, df = 6, *p* < 0.0001, AIC = 316.43.

Variable	OR	95% CI	*p*-Value
Diabetic Status	2.04	0.88–4.76	0.0981
Age	1.02	0.99–1.04	0.213
BMI	1.01	0.97–1.04	0.717
SHR_z3	1.33	0.74–2.40	0.342
HbA1c	1.04	0.86–1.25	0.705
RX_1 (admission severity)	2.31	1.61–3.31	<0.0001

**Table 4:** Adjusted for Rx_1 and comorbidities count; Overall model: χ^2^ = 62.521, df = 7, *p* < 0.0001; AIC = 305.97.

Variable	OR	95% CI	*p*-Value
Diabetic Status	1.37	0.56–3.39	0.490
RX_1	2.12	1.47–3.07	0.0001
Comorbidity Count	1.45	1.17–1.79	0.0006

### 3.6. Sensitivity Analyses for Attrition

The between-group difference in day 6 radiological severity was robust to the handling of patients lost to follow-up. In the primary survivor analysis, diabetic patients retained a higher residual severity than non-diabetic patients (median Rx 1.0 [n = 188] vs. 0.0 [n = 167]; Mann–Whitney U = 22,151.5, *p* < 0.0001). In the worst-case analysis, in which the 17 early deaths (14 diabetic, 3 non-diabetic) were assigned the maximal day 6 score (Rx = 3), the diabetic disadvantage widened: the diabetic median remained 1.0 (n = 202) versus 0.0 in non-diabetics (n = 170), U = 24,513.0, *p* < 0.0001. In the best-case analysis, in which the low-severity early departures lost through DAMA (9 diabetic, 49 non-diabetic) were assigned a day 6 score of 0, the difference narrowed but remained significant and in the same direction (diabetic median 1.0 [n = 197] vs. 0.0 [n = 216]; U = 30,509.5, *p* < 0.0001). The composite adverse endpoint (death, persistent radiological abnormality at day 6, or failure to improve), evaluated across all patients with a known day 6 status, was reached by 135 of 202 diabetic patients (66.8%) compared with 44 of 170 non-diabetic patients (25.9%) (odds ratio 5.77, 95% CI 3.67–9.06; χ^2^ = 60.4, *p* < 0.0001). These analyses indicate that the radiological lag is not an artifact of selective survival and that, if anything, the survivor-only comparison is conservative, since the concentration of early mortality among high-severity diabetic cases means the survivor-restricted estimate under-states rather than overstates the true burden in the diabetic group.

## 4. Discussion

The primary finding of this prospective study is the identification of a radiological lag in diabetic patients hospitalized with viral pneumonia [15,20,21,22,23,24]. Our data supports a disadvantaged starting point hypothesis: diabetic patients arrive with nearly double the radiological severity of non-diabetics. Although they heal at a pace identical to the control group, they exhibit persistent radiographic evidence of lung pathology by the end of the first week of medical care. The higher admission severity observed in diabetics (Median 2.0 vs. 1.0) suggests that the diabetic state predisposes patients to more rapid initial parenchymal damage, creating a disadvantage that persists despite standard healing rates. While our cohort included a diverse range of viral pathogens, the observed radiological lag was identified as a generalized metabolic response to viral pulmonary injury in diabetics, rather than a pathogen-specific phenomenon.

Our unified multivariable regression model confirms this dynamic by showing that once the baseline diabetic state is established, patients face a 2.64-fold increase in the odds of delayed radiographic clearance, independent of age, body mass index, or variations in acute phase glycemic metrics (SHR; Table 2 and Table 3). As shown in the subsequent adjusted models (Table 3 and Table 4) and discussed below, this association is substantially mediated by baseline severity and chronic comorbidity burden, suggesting that the diabetic state operates upstream of acute clearance kinetics rather than through an intrinsic effect on healing rate. This statistical finding is consistent with, though does not definitively establish, a binary-threshold interpretation, in which the established metabolic state of diabetes may exert a more uniform effect on lung tissue repair than do short-term glycemic fluctuations. The observed radiological lag may be attributed to the impaired macrophage phagocytosis and prolonged pro-inflammatory cytokine signaling typical of the diabetic pulmonary environment [11,12,14,25,26,27,28,29], which prevents the rapid clearance of viral cellular debris even as systemic inflammation (CRP) begins to subside [30,31,32,33].

This lag is clinically significant, as evidenced by the high correlation between radiological worsening and the need for HFNO support or OTI. The lack of significant metabolic predictors within the regression models suggests that this lag may represent a generalized feature within the hospitalized diabetic cohort for viral pneumonia, rather than a factor of individual glucose levels during hospitalization. The radiological lag is strongly associated with an increased length of hospital stay in the diabetic cohort, as discharge was often contingent upon the physical resolution of parenchymal infiltrates.

The selection of a 6-day evaluation window serves as a critical measure of clinical readiness, since protocol-based treatments for major viral pathogens typically conclude by day 5, the day 6 scan reveals a significant disparity: while non-diabetics are often radiologically cleared and ready for discharge (Median Rx: 0.0), diabetics remain in a state of persistent pathology (Median Rx: 1.0). This confirms that the metabolic burden of diabetes extends the required recovery time beyond standard treatment windows, regardless of the pathogen involved [17,22,34].

The observed radiological lag translates directly into increased healthcare utilization. Diabetic patients in this cohort required significantly longer hospital stays compared to non-diabetics. This prolonged hospitalization is physically evidenced by the delayed clearance of pulmonary infiltrates. The radiological progression observed in a subset of patients with diabetes consistently preceded the clinical requirement for High-Flow Nasal Oxygen, underscoring that the 0–3 scoring system used in this prospective study serves as a viable prognostic tool for clinical escalation. All 11 diabetic patients (100%) who exhibited early radiological progression on our ordinal scale ultimately required clinical escalation to advanced respiratory support (HFNO or mechanical ventilation). Although this correlation is striking, it is based on a small number of patients (n = 11) and should be regarded as hypothesis-generating; the prognostic value of tracking early dynamic changes on this scale will require formal validation, including prospective calibration against established severity indices, in larger cohorts before it can be recommended for routine clinical use.

A critical strength of this study is the comprehensive analysis of patient attrition. Longitudinal clinical studies frequently encounter survivor bias, where the most severe cases are omitted from longitudinal analysis due to early mortality. By identifying the lethal split, we support that diabetic attrition was primarily driven by mortality (60.9%) rather than the low-severity early departures (DAMA) that dominated attrition in non-diabetics (94.2%). Sensitivity analyses (Section 3.6) accordingly suggest that the radiological lag observed among surviving diabetics likely underestimates the true disease burden in this population.

While diabetes status was a predictor of severity, individual glycemic metrics (HbA1c and SHR_z3) failed to predict the rate of resolution within the diabetic cohort. One interpretation consistent with these data is that the established metabolic environment of diabetes, rather than short-term fluctuations in acute glucose, is the more relevant correlate of impaired clearance kinetics over the first week of treatment. This should be regarded as a hypothesis rather than a demonstrated mechanism: the absence of an association with HbA1c and SHR_z3 may also reflect limited statistical power for these continuous predictors, the narrow follow-up window, or unmeasured confounding, and a graded (rather than strictly binary) relationship cannot be excluded.

These adjusted analyses reframe the diabetic radiological lag less as an independent metabolic phenomenon and more as a downstream consequence of the higher baseline severity and comorbidity burden with which diabetic patients present. This is consistent with the disadvantaged starting point hypothesis: diabetes operates upstream, conditioning a sicker presentation, after which healing kinetics in survivors are broadly comparable.

This prospective study provides robust data, certain limitations remain. The six-day follow-up window, while clinically standard for acute viral monitoring, may be too short to capture the full resolution of bronchopneumonia in the most severe diabetic cases. Additionally, the high mortality rate in the diabetic attrition group necessitated a focus on survivors for the longitudinal analysis, though this was statistically addressed through separate attrition modeling.

Several limitations should be acknowledged. First, the regression model did not exhaustively adjust for all potential confounders; in particular the burden of chronic comorbidities and any subtle differences in supportive treatment could each influence the trajectory of radiological resolution, and residual confounding cannot be excluded despite the standardization of corticosteroid exposure. Second, the 0–3 ordinal scale was developed internally for parsimony and clinical applicability. Although both films were scored by two independent readers with consensus resolution of disagreements, formal inter-reader reliability statistics were not computed in the present study; this represents a methodological limitation that we plan to address in a forthcoming validation analysis. The scale has also not been externally validated against quantitative or volumetric imaging measures, and its generalizability to other settings remains to be established. Third, the study was conducted at a single tertiary infectious-diseases center, which may limit external validity and introduce referral bias toward more severe presentations. Finally, because the cohort comprised a heterogeneous group of viral pathogens, the analysis was not powered to detect pathogen-specific differences in resolution. These limitations temper the strength of the conclusions and reinforce the need for external, multicenter validation.

## 5. Conclusions

In our single-center cohort, the data suggest that diabetic patients hospitalized with viral respiratory infections experience a distinct radiological lag, characterized by higher baseline severity and delayed pulmonary clearance. The identification of the lethal split highlights a clinical vulnerability within this specific population, suggesting that study attrition among diabetic patients may be heavily influenced by early mortality rather than the low-severity early departures that characterized non-diabetic attrition. Adjusted analyses suggest that this vulnerability operates primarily through higher presenting severity and chronic comorbidity burden rather than through an independent acute metabolic mechanism, identifying these upstream factors as the most promising targets for early risk stratification. These preliminary findings point toward a potential need for intensified early monitoring and personalized respiratory management, potentially incorporating targeted metabolic modulators [11,12,35], to mitigate the localized physical and systemic burden of viral pneumonia. Ultimately, by quantifying this radiological lag and identifying the lethal split within our study population, this work proposes a framework connecting biochemical metabolic dysfunction to physical clinical reality, offering a promising foundation for early clinical escalation strategies that warrants further validation in larger, multicenter trials. Finally, our data suggest that clinicians should exercise caution when relying solely on the normalization of biochemical markers in diabetic patients, as physical lung resolution may lag behind, potentially creating a deceptive window of apparent recovery while the patient remains radiologically compromised. Future work should externally validate the ordinal scoring system against quantitative imaging, extend the follow-up horizon to capture complete resolution in the most severe cases, and prospectively test whether early radiological monitoring improves the timing of respiratory escalation and discharge in diabetic patients across multiple centers and defined pathogen subgroups.

## Data Availability

Data availability is subject to hospital approval (due to internal regulations of the hospital—Regulation UE nr. 679 from 2016 regarding protection of personal data).

## Abbreviations

The following abbreviations are used in this manuscript:

ARDS	Acute Respiratory Distress Syndrome
AUC	Area Under the Curve
BMI	Body Mass Index
CI	Confidence Interval
CRP	C-Reactive Protein
DAMA	Discharged Against Medical Advice
HbA1c	Glycated Hemoglobin
hMPV	Human Metapneumovirus
IQR	Interquartile Range
OR	Odds Ratio
OTI	Orotracheal Intubation
PCR	Polymerase Chain Reaction
ROC	Receiver Operating Characteristic
RSV	Respiratory Syncytial Virus
Rx	Radiological Severity Score/Parenchymal Involvement
SARS-CoV-2	Severe Acute Respiratory Syndrome Coronavirus 2
